# New FDA safety warnings for LABAs: A call for asthma guidelines revisit for solo beta agonist

**DOI:** 10.4103/1817-1737.62468

**Published:** 2010

**Authors:** Emad A. Koshak

**Affiliations:** *Albaha University, Albaha, Saudi Arabia*

The important paradigm shift for asthma care that focuses on clinical control has revolutionized the understanding of the disease and its optimal pharmacological care.[1] Unfortunately, the use of any pharmacological agent is hardly free from any side effects or health hazards, the details regarding which need to be continuously updated for health care workers.

The February 2010 new release from the U. S. Food and Drug Administration (FDA) requires new safety measures and warnings for the use of inhaled long-acting beta agonists (LABAs) in asthma control [Table 1].[2] Such warnings originated from several clinical trials that demonstrated that asthmatics taking inhaled LABAs without the use of other add-on asthma controller medications such as inhaled corticosteroids (ICs) faced an augmented rate of asthma exacerbations, hospitalization and death.[2] A perspective has also been published clarifying further these FDA warnings.[3]

**Table 1:** New FDA safety warnings of label changes for LABAs

Single-ingredient LABAs should only be used in combination with an asthma controller medication; they should not be used alone. LABAs should only be used long-term in patients whose asthma cannot be adequately controlled on asthma controller medications. LABAs should be used for the shortest duration required to achieve control of asthma symptoms and discontinued, if possible, once asthma control is achieved. Patients should then be maintained on an asthma controller medication. Pediatric and adolescent patients who require the addition of a LABA to an inhaled corticosteroid should use a combination product containing both an inhaled corticosteroid and a LABA, to ensure compliance with both medications. Adopted from the U. S. FDA website[2]

Certainly, this new FDA safety release on the use of LABAs will create some confusion among asthma care workers, patients and related drug companies. However, the bright side of such warnings is that it will for sure reinforce the tendency of asthma care workers to revisit the stepwise (whether up or down) approach of pharmacotherapy recommended by most recent national and international asthma guidelines.[4–6] The main theme of these guidelines is to achieve total or near-total control of asthma symptoms with the use of the least number and doses of medications because of their cost and potential side effects.

Some of these FDA warnings for LABA inhalers can be considered as fine-tuning and reinforcement of the already comparable recommendations stated in most recent asthma guidelines.[4–6]

1. The use of LABAs as monotherapy and as a reliever medication is strongly discouraged because of potential health hazards.
2. LABAs may be used in combination with ICs in the stepping up of treatment for the long-term management of only such asthma that is uncontrolled by controller medications.
3. Patients should be maintained on an asthma controller medication once asthma control is achieved.
4. A combination product of ICs and LABAs should be used only to ensure compliance with both medications.

Surprisingly, among these FDA warnings, the new recommendations are to shorten the period of use of LABAs even if in combination with ICs and to discontinue its use, if possible, once asthma control is achieved. These new advices are somewhat welcome as true phenomena of stepping-down in the treatment of well-controlled asthma.

These recommendations might open the gate for the use of other alternative controller agents that can optimize asthma control. They have also stressed the implementation of non-pharmacological measures for achieving the control of asthma. These include the identification and avoidance of triggering and exacerbating factors (including inhalant irritants and allergens) to minimize the overall use of any unnecessary pharmacological agents in order to avoid their potential future hazards for health.[7] These measures are frequently ignored although they are at the base of asthma management pyramid in all asthma guidelines.[4–6]

Furthermore, I would also like to take these warnings to share a personal view about similar safety requirements that may be needed for the solo use of short-acting beta agonists (SABAs), which is recommended in asthma guidelines as reliever inhalers.[4–6] Such concerns have originated from some previously published reports focused on the potential health hazards and mortality from the overuse of solo SABAs.[7] Hence I would like to propose this question: Should we be thinking to avoid the use of any solo beta-agonist inhaler in asthma management? Obviously, the conclusive answers to this question will need a lot of research work to explore the benefit/ risk profile of this innovative view in asthma pharmacological management.

In conclusion, this new FDA safety release on the warnings for the use of inhaled LABAs is very well saluted. It will motivate the asthma care workers to revisit the stepwise pharmacotherapy approach recommended by most current asthma guidelines. It recommends the use of LABAs to be limited to the combination with ICs for cases with uncontrolled asthma and to be administered for the shortest possible duration. The solo use of LABAs might soon become history in the management of asthma. Extension of similar cautions for solo use of SABAs is a personal vision that will be challenged further by benefit/ risk profile in future clinical investigations.
